# Preliminary Study of Dynamic Cerebral Autoregulation in Acute Ischemic Stroke: Association With Clinical Factors

**DOI:** 10.3389/fneur.2018.01006

**Published:** 2018-11-26

**Authors:** Hongyin Ma, Zhen-Ni Guo, Hang Jin, Xiuli Yan, Jia Liu, Shan Lv, Peng Zhang, Xin Sun, Yi Yang

**Affiliations:** ^1^Stroke Center, Department of Neurology, the First Hospital of Jilin University, Changchun, China; ^2^Neuroscience Center, Department of Neurology, The First Hospital of Jilin University, Changchun, China; ^3^Shenzhen Institutes of Advanced Technology, Chinese Academy of Sciences, Shenzhen University Town, Shenzhen, China

**Keywords:** dynamic cerebral autoregulation, transfer function analysis, acute ischemic stroke, clinical factors, rt-PA thrombolysis

## Abstract

**Background and Purpose:** Dynamic cerebral autoregulation (dCA) is probably impaired in the acute and even subacute phases after acute ischemic stroke (AIS); however, the relationship between relevant clinical factors and dCA after AIS has not been investigated. The identification of possible determinants may therefore provide potential therapeutic targets to improve dCA in AIS.

**Methods:** This study enrolled 67 consecutive patients diagnosed with AIS within 3 days from symptom onset. Serial measurements were performed 1–3 days (measurement 1) and 7–10 days (measurement 2) after the onset. Middle cerebral artery blood flow velocities and simultaneous arterial blood pressure (ABP) were recorded continuously with transcranial Doppler combined with a servo-controlled finger plethysmograph. Transfer function analysis was used to derive dCA parameters, phase difference (PD), and coherence in low-frequency range (0.06–0.12 Hz). Univariate and multivariate linear regression analyses were conducted to determine the relationship between clinical factors and PD within the two time points of measurements. Multivariate logistic regression was performed to reveal the relationship between PD and clinical outcomes.

**Results:** Bilateral PD was significantly lower (indicating impaired dCA) in AIS patients, both in measurement 1 and measurement 2 when compared with those of healthy controls (all *P* < 0.001). After controlling for relevant clinical factors, in measurement 1, age (β = −0.29, *P* = 0.01), recombinant tissue plasminogen activator (rt-PA) intravenous thrombolysis (β = 0.25, *P* = 0.034), subtype of large-artery atherosclerosis (LAA) (β = −0.31, *P* = 0.007), and uric acid level (β = −0.32, *P* = 0.009) were significant independent predictors of ipsilateral PD. In measurement 2, subtype of LAA (β = −0.28, *P* = 0.049) and uric acid level (β = −0.43, *P* = 0.005) were still significant predictive values for ipsilateral PD. After adjusting for age and National Institutes of Health Stroke Scale at admission, ipsilateral PD >35.37° in measurement 2 was independent predictor of good clinical outcomes (adjusted OR = 6.97, 95% CI: 1.27–38.14, *P* = 0.03).

**Conclusion:** DCA was sustained to be bilaterally impaired in the acute and even subacute phase after AIS. Patients who receiving rt-PA thrombolysis tended to have a better dCA in the acute phase. Increasing age, subtype of LAA, and higher uric acid level had prognostic value for disturbed autoregulation. A relatively preserved dCA may predict good clinical outcomes.

## Introduction

Cerebral autoregulation (CA) was initially considered as an intrinsic protective mechanism of the brain, which ensured relatively constant cerebral blood flow (CBF) despite fluctuations in arterial blood pressure (ABP) or cerebral perfusion pressure (1). However, this capability may be impaired or even vanish after ischemic stroke; this means that under this pathologic circumstance, the brain tissue may have the risk of hypo- or hyperperfusion once encountering inappropriate ABP, leading to further damage of the brain and thus deterioration of neurological functions and clinical outcomes (2–5). Thus, CA is important when making individualized clinical decisions in acute ischemic stroke (AIS) patients.

Dynamic cerebral autoregulation (dCA) measurement, with non-invasive real-time continuous recording of CBF and ABP, usually performed by transcranial Doppler (TCD) in combination with continuous blood pressure measurement, provided a completely new notion of CA (6). This method could allow more access to the dynamic characteristics of CA with minimized interference and stimulus; thus, it is prevalent in studies on cerebral vascular diseases and is expected to be widely applied in clinical practice (7). Currently, most previous studies have agreed that dCA is probably impaired in acute and even subacute phases after ischemic stroke (3–5, 8–13); at the same time, inter-individual difference in autoregulation capability among patients has also been noted. Although some dCA studies revealed that the discrepancy in individuals may partially be explained by different mechanisms of pathogenesis (8, 9), characteristics of infarction (3, 4, 10), and even temporal courses of disease (11–13), few studies have comprehensively clarified the relationship between clinical factors and impaired dCA. Further, screening prognostic information of dCA is significant for deep understanding of the mechanisms underlying impaired autoregulation in AIS patients and possibly provides potential therapeutic targets in the future.

Thus, in the present study, we aimed to investigate the relationship between specific clinical factors and autoregulation and attempted to provide noteworthy clues for further dCA studies in AIS patients.

## Materials and methods

### Subjects and controls

This prospective observational study was approved by the Ethics Committee of the First Hospital of Jilin University, China, and written informed consent was obtained from all subjects or their direct relatives. The study enrolled 67 consecutive AIS patients admitted to the Comprehensive Stroke Center, Department of Neurology, First Hospital of Jinlin University, China, from October 2016 to September 2017. Patients were diagnosed with AIS according to clinical symptoms and the findings of physical examination, brain computed tomography, and/or magnetic resonance imaging, as well as, routine laboratory tests. This study included AIS patients who met the following criteria: (1) unilateral middle cerebral artery territory infarction; (2) admitted to hospital within 3 days after symptom onset; (3) sufficient bilateral temporal bone window for insonation; and (4) conscious and cooperating for dCA measurements. Patients were excluded if they: (1) underwent any endovascular interventions after stroke, including intra-arterial fibrinolysis, mechanical clot retrieval, acute angioplasty, or stenting; (2) suffered from stroke within 3 months; (3) suffered from more than 70% stenosis/occlusions of intracranial or/and extracranial major artery on the contralateral side of infarction; (4) were accompanied with atrial fibrillation, myocardial infarction, heart failure, severe anemia, or hyperthyroidism as diagnosed by two physicians with electrocardiogram and laboratory tests, which may undermine the stability of hemodynamics; (5) stroke of cardioembolism and other determined cause grouped by Trial of ORG 10172 in Acute Stroke Treatment (TOAST) criteria (14), such as cerebral vessel dissection and vasculitis; and (6) took anti-anxiety medication. All subjects in the Comprehensive Stroke Center received routine medical treatment and general stroke care according to early management guideline of AIS patients (15).

Data of 60 age- and sex-matched non-smoking healthy volunteers were obtained from our database as a reference value of dCA. They also underwent brain CT/MRI and vascular imaging screening (TCD and carotid ultrasound) before dCA measurement to exclude prior cerebral vascular disease and major intracranial and/or extracranial vascular stenosis/occlusion.

### Clinical data collection

Historical information was extracted from interview and previous medical records. Neurological examination and National Institutes of Health Stroke Scale scores were timely performed once upon admission. Brain computed tomography, and/or magnetic resonance imaging were performed within 3 days after symptom onset. TCD, carotid ultrasound, and magnetic resonance angiography were routinely performed to evaluate occlusions/stenosis of intracranial and extracranial vessels. Twenty-four hour Holter monitoring was used to identify atrial fibrillation and other arrhythmias. Laboratory results, including low-density lipoprotein cholesterol, homocysteine, glycosylated hemoglobin A (1C), and uric acid level were recorded. Stroke location was classified as cortical, subcortical, or both cortical and subcortical lesions extracted from brain imaging data by two neuroradiologists. Subtype of AIS was determined as large-artery atherosclerosis (LAA), cardioembolism, small-artery occlusion (SAO), other determined causes, and undetermined etiology (UE) according to the TOAST classification (14) after comprehensively evaluating clinical features, risk factors, and results of diagnostic tests by two neurologists. Receipt of intravenous recombinant tissue plasminogen activator (rt-PA) thrombolysis was also recorded. Modified Rankin Scale (mRS) was performed after 3 months follow up since stroke onset by a neurologist and 0–2 points of mRS was defined as good functional outcome.

### DCA measurement

DCA measurement was conducted in a quiet laboratory room with external stimuli minimized. Each subject underwent dCA measurement at two time points: 1–3 days and 7–10 days after symptoms onset. A specialized neurovascular ultrasound doctor performed all measurements. The subjects were instructed to avoid alcohol, caffeine, and nicotine consumption for at least 12 h. Before measurements, the subjects adopted a relaxed supine position for more than 10 min, and baseline blood pressure was then measured at the left brachial artery (automatic blood pressure monitor, Omron 711). Bilateral middle cerebral artery was probed through bilateral temporal bone window at a depth of 45–60 mm with 2 MHz probes using TCD (EMS-9PB, Delica, China). A custom-made frame with two probes was then fixed for recording non-invasive cerebral blood flow velocity (CBFV). Spontaneous ABP was simultaneously recorded using a servo-controlled plethysmograph (Finometer Pro, The Netherlands) on the middle finger with an appropriate finger cuff size positioned at the heart level. After steady signals of ABP and CBFV were established, real-time recording was started for at least 10 min. The recorded data were then stored for further dCA analysis.

### Analysis of DCA

The recorded data were processed with a personal computer using MATLAB (MathWorks, Natick, MA, USA), as we previously reported (16). Beat-to-beat alignment of the data was performed using a cross-correlation function to remove possible time lags. A 3rd-order Butterworth low-pass filter (cutoff at 0.5 Hz) was then applied as an anti-aliasing filter before down-sampling the data to 1 Hz. DCA parameters were derived using the transfer function analysis between ABP and CBFV, which was calculated as the quotient of the cross-spectrum of the two signals. PD was derived in low-frequency range (0.06–0.12 Hz), in which dCA was proven to operate efficiently (17). PD was not available for further statistical analysis if frequent signal artifacts were present in raw data of CBFVs or ABP and if derived coherence of transfer function was < 0.5.

### Statistical analysis

All data were analyzed by Statistical Program for Social Sciences version 21.0 (SPSS, IBM, West Grove, PA, USA). For continuous variables, the Kolmogorov–Smirnov test was used to test for the normality of the data. The difference between control and AIS patients at each measurement was compared with Student's 2-sample *t*-test as data were normally distributed; differences in AIS patients between measurements were examined using a paired Student's *t*-test. Categorical variables were reported by the rate or constituent ratio. The chi-squared test was used to compare the difference between categorical variables. Univariate analyses were conducted to identify the correlation between clinical factors and bilateral PD on each measurement. Multivariate linear regression analyses were conducted to associate clinical factors with ipsilateral PD of each measurement, respectively. In the multivariate linear regression model of measurement 1 and 2, the dependent variable was ipsilateral PD in each measurement, independent variables were demographic and clinical factors including age, gender, administration of rt-PA thrombolysis, stroke subtype, uric acid level, and NIHSS on admission. Independent variables were selected out of clinical consideration, as well as, the results of univariate analyses as reference. Spearman's rho correlation analysis was used to correlate PD of each measurement and mRS at 3 months. A receiver operating characteristic (ROC) curve was generated to determine the optimal cut-off point of PD associated with outcomes, and then multivariate logistic regression was performed to reveal the relationship between PD and outcomes. Statistical tests were 2-tailed, and *P* < 0.05 was considered statistically significant.

## Results

Among all 67 enrolled AIS patients, 110 segments of measurement were successfully performed, and derived indices of dCA were available for further statistical analysis. Sixty-four and 46 measurements were performed on 1–3 and 7–10 days, respectively, after stroke onset. Twelve scheduled measurements were unavailable for the patients who had been discharged, and 12 sets of derived indices were not available for further statistical analysis because of frequent signal artifacts (*N* = 6) and low coherence value of transfer function (*N* = 6). Sixty age- and sex-matched non-smoking healthy controls (53.4 ± 10.3 years, 49 males) were also collected. Demographic and clinical information of involved patients is presented in Table 1. Hemodynamic status during dCA measurements is presented in Table 2.

**Table 1:** Demographic and clinical information of AIS patients.

	**AIS patients (*N* = 67)**
Age, year	52.8 ± 10.2
Male	56 (83.6)
Smoking	45 (67.2)
Hypertension	33 (49.3)
Diabetes mellitus	13 (19.4)
Previous symptomatic stroke	8 (11.9)
Stroke location	
Cortical	5 (7.5)
Subcortical	56 (83.6)
Both cortical and subcortical	6 (9.0)
Toast classification	
LAA subtype	12 (17.9)
SAO subtype	47 (70.1)
UE subtype	8 (11.9)
Rt-PA thrombolysis	39 (58.2)
NIHSS at admission, points	6.0 ± 3.3
LDL-C, mmol/L	2.9 ± 0.8
HbA1c, %	6.2 ± 1.5
Homocysteine, μmol/L	20.6 ± 18.7
Uric acid, mmol/L	334.9 ± 94.8

*Data are expressed as mean ± SD or absolute number (percentage) where appropriate. AIS, acute ischemic stroke; TOAST, the Trial of Org 10172 in Acute Stroke Treatment; LAA, large-artery atherosclerosis; SAO, small artery occlusion; UE, undetermined etiology; Rt-PA, recombinant tissue plasminogen activator; NIHSS, National Institutes of Health Stroke Scale; LDL-C, low density lipoprotein cholesterol; HbA1c, glycosylated hemoglobin A (1C)*.

**Table 2:** Hemodynamic status during dCA measurements.

	**Measurement 1 (Days 1–3)**	**Measurement 2 (Days 7–10)**	**Controls**
MAP, mmHg	109.3 ± 11.2^*^	106.7 ± 12.8^*^	92.1 ± 11.3
HR, beats/min	70.5 ± 9.3	71.0 ± 11.6	73.7 ± 11.4
MCA CBFV, cm/s			
Ipsilateral side	58.3 ± 16.3	57.0 ± 11.4	61.0 ± 8.7
Contralateral side	61.4 ± 9.7	60.8 ± 7.6	60.8 ± 7.6
EtCO_2_, mmHg	36.0 ± 1.3	35.7 ± 0.9	35.8 ± 1.0

*DCA, dynamic cerebral autoregulation; MAP, mean arterial blood pressure; HR, heart rate; MCA, middle cerebral artery; CBFV, cerebral blood flow velocity; EtCO_2_, end tidal CO_2_*.

* *P < 0.05 compared with healthy controls*.

### DCA in AIS patients vs. controls

**Figure 1:**
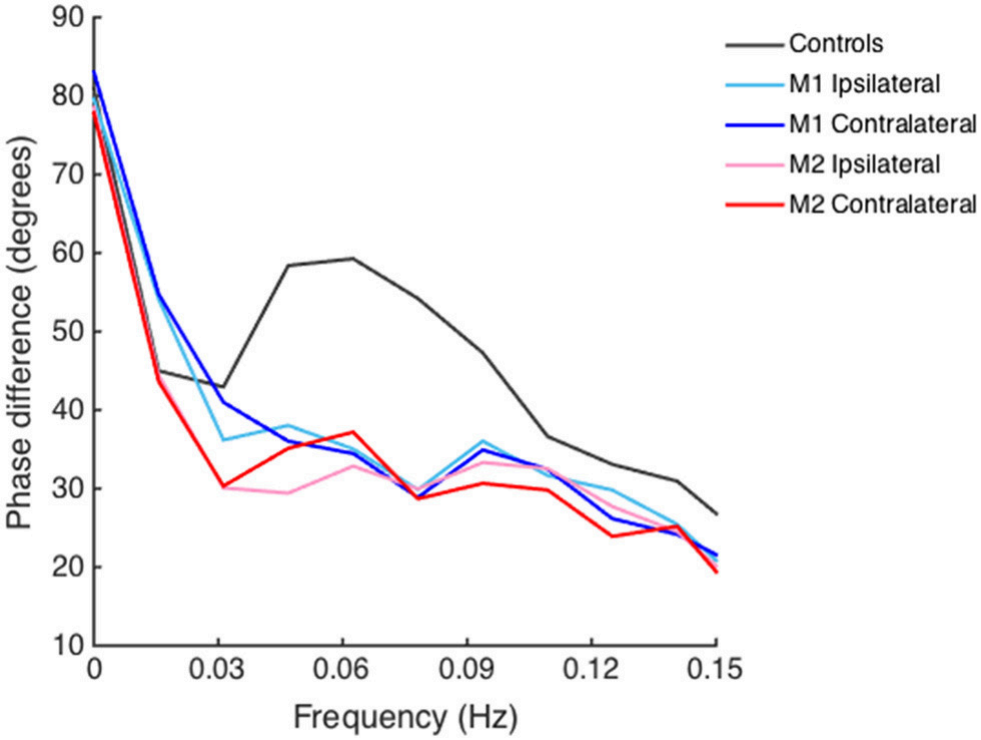
Phase difference of acute ischemic stroke (AIS) patients and healthy controls derived from transfer function analysis in frequency domain. Lines depicted with different colors denote phase difference on the ipsilateral and contralateral side of AIS patients and healthy controls. Bilateral decreased phase difference is shown in both measurement 1 (M1) and measurement 2 (M2) in AIS patients as compared to that in healthy controls in low-frequency range (0.06–0.12 Hz).

PD did not differ between the ipsilateral and contralateral side both in measurement 1 (34.3 ± 17.8° vs. 34.2 ± 16.7°, *t* = 0.09, *P* = 0.93) and measurement 2 (35.2 ± 19.3° vs. 32.2 ± 16.8°, *t* = 1.53, *P* = 0.13). Bilateral PD was significantly lower (indicating impaired dCA) in AIS patients both in measurements 1 and 2 than in healthy controls (55.8 ± 11.5°, all *P* < 0.001). There were no appreciable differences in PD between the two time points of measurements in both ipsilateral (*t* = 0.18, *P* = 0.86) and contralateral side (*t* = 0.93, *P* = 0.36). Bilateral PD in frequency domain of AIS patients and healthy controls is depicted in Figure 1.

### Clinical and laboratory factors associated with PD in AIS patients

*P*-values for univariate analyses of PD and clinical factors are showed in Table 3. There was no significant correlation between PD of any side and the following factors in each measurement: gender, history of smoking, hypertension, diabetes mellitus, previous symptomatic stroke, stroke location, NIHSS on admission, low density lipoprotein cholesterol, glycosylated hemoglobin A (1C), homocysteine level. Patients who received intravenous rt-PA thrombolysis showed a significantly higher ipsilateral PD (indicating more effective dCA) than those did not (38.5 ± 17.0° vs. 28.9 ± 17.7°, *t* = 2.17, *P* = 0.033) in measurement 1. Subtype of LAA patients had a significantly lower PD (indicating inferior dCA) on the ipsilateral side than those of SAO or UE subtype in measurement 1 (LAA vs. SAO vs. UE, 23.0 ± 18.8° vs. 37.1 ± 17.1° vs. 35.8 ± 15.3°, *F* = 3.18, *P* = 0.049). PD difference within stroke subtypes still existed in measurement 2 especially on contralateral side (LAA vs. SAO vs. UE: 18.7 ± 12.4° vs. 33.0 ± 15.6° vs. 43.7 ± 19.6°, *F* = 4.24, *P* = 0.021). Moreover, higher uric acid level was associated with lower bilateral PD in both measurements, and this factor remained significant in the following multivariate analyses.

**Table 3:** *P*-value for univariate analyses of PD and clinical factors.

	**PD**			
**Clinical factors**	**Measurement 1**		**Measurement 2**	
	**Ipsilateral**	**Contralateral**	**Ipsilateral**	**Contralateral**
Age	0.021^*^	0.054	0.333	0.048^*^
Gender	0.184	0.677	0.467	0.311
Smoking	0.475	0.814	0.473	0.156
Hypertension	0.647	0.655	0.612	0.211
Diabetes mellitus	0.308	0.164	0.330	0.144
Previous symptomatic stroke	0.683	0.862	0.477	0.416
Stroke location	0.120	0.785	0.068	0.065
Stroke subtype	0.049^*^	0.365	0.073	0.021^*^
Rt-PA thrombolysis	0.033^*^	0.235	0.632	0.617
NIHSS at admission	0.408	0.740	0.380	0.925
LDL-C	0.906	0.690	0.512	0.884
HbA1c	0.648	0.465	0.160	0.054
Homocysteine	0.380	0.515	0.960	0.656
Uric acid	0.026^*^	0.027^*^	0.010^*^	0.022^*^

*PD, phase difference; Rt-PA, recombinant tissue plasminogen activator; NIHSS, National Institutes of Health Stroke Scale; LDL-C, low density lipoprotein cholesterol; HbA1c, glycosylated hemoglobin A (1C)*.

* *P < 0.05 in according univariate analysis*.

Multivariate linear regression models of ipsilateral PD are summarized in Table 4. In the model of measurement 1, age, rt-PA thrombolysis, subtype of LAA, and uric acid level were significant independent predictors for ipsilateral PD; in measurement 2, subtype of LAA and uric acid level remained significant as predictive values for ipsilateral PD.

**Table 4:** Multivariate linear regression models of ipsilateral PD.

**Factors**	**Standard β**	***P-*Value**
**MEASUREMENT 1**		
Age	−0.29	0.010^*^
Gender	0.01	0.925
Rt-PA thrombolysis	0.25	0.034^*^
Stroke subtype		
SAO	Reference
LAA	−0.31	0.007^*^
UE	−0.07	0.555
Uric acid	−0.32	0.009^*^
NIHSS on admission	0.05	0.653
**MEASUREMENT 2**		
Age	−0.20	0.144
Gender	−0.09	0.532
Rt-PA thrombolysis	0.21	0.147
Stroke subtype		
SAO	Reference
LAA	−0.28	0.049^*^
UE	0.12	0.436
Uric acid	−0.43	0.005^*^
NIHSS on admission	−0.15	0.298

* *Significant factors according to multivariate regression models. PD, phase difference; Rt-PA, recombinant tissue plasminogen activator; SAO, small artery occlusion; LAA, large-artery atherosclerosis; UE, undetermined etiology; NIHSS, National Institutes of Health Stroke Scale*.

Three months mRS of all the patients was successfully collected. The median mRS score was 1 (range 0–6). Higher ipsilateral PD in measurement 2 was correlated with lower mRS (*r* = −0.359, *P* = 0.014), indicating better functional outcomes. ROC curve was generated to determine the cut-off point that optimized the sensitivity and specificity associated with clinical outcomes. The cut-off point of PD for predicting clinical outcomes was 35.37°, which yielded a specificity of 83% and a sensitivity of 59%. The ROC curve analysis revealed that the area under the curve (AUC) was 0.72 (95% CI: 0.55–0.90). After adjusting for age and NIHSS at admission, ipsilateral PD>35.37° in measurement 2 was an independent predictor of good clinical outcomes (adjusted OR = 6.97, 95% CI: 1.27–38.14, *P* = 0.03).

## Discussion

Our study presented a series of dCA measurements targeting AIS patients with relatively comprehensive clinical data and attempted to identify possible determinants of autoregulation after stroke. We found dCA was bilaterally impaired in acute phase (1–3 days), and this tendency was sustained for at least 7–10 days after AIS. Patients who receiving rt-PA thrombolysis tended to have a better dCA in acute phase. After controlling for demographic and relevant clinical factors, increasing age, subtype of LAA, and higher uric acid level had the prognostic value for disturbed autoregulation. A relatively preserved dCA may predict good clinical outcomes.

Although studies to date concluded that dCA was impaired in AIS (3–5, 8–13, 18, 19), the characteristics of impaired dCA have not been fully investigated. It remains controversial whether dCA is focally or globally impaired with unilateral infarct lesion (19). Some studies considered that it may differ according to various stroke subtypes (8–10, 19). For the subtype of SAO or lacunar infarction, dCA was considered bilaterally impaired; however, for the LAA subtype, the conclusions were drawn differently. Some studies indicated that impaired dCA was more evident on the ipsilateral hemisphere, while others considered that there was a bilateral impairment of dCA; Reinhard et al. observed dCA had the tendency to worsen on the ipsilateral side and spread to the contralateral side during the first days after AIS (3, 20). Our results supported the bilateral impairment of dCA within the involved stroke subtypes. By evaluating the detailed data for trends of dCA in different subtypes between the two time points of measurement, it was not difficult to find that PD in LAA subtype was lower than that in the other two groups on 1–3 days and even became worse over 7–10 days bilaterally; this finding was consistent with the findings of Reinhard et al. Unlike LAA, we were surprised to note the fast recovery of PD in UE subtype from 1–3 to 7–10 days, but PD in the SAO subtype remained constantly disturbed. The pathology of SAO was characterized by cerebral small vessel atherosclerosis with accumulating insult due to various risk factors; therefore, even with unilateral lacunar infarction, patients usually had bilateral disturbed dCA in different degrees, and this disturbance could be sustained as long as 6-month follow-up, as reported by Guo et al. (18). Clinically, the UE subtype was identified as neither lacunar strokes nor due to atherosclerosis without a detectable cardioembolic source. Furthermore, persuasive evidence showed that most UE patients were thromboembolic of potential low-risk embolic sources (21), and embolic obstructions usually spontaneously recanalize, which may explain the restorable characteristics of dCA in this group.

The relationship between rt-PA thrombolysis and dCA has not been frequently studied. Theoretically, rt-PA thrombolysis may have beneficial effects on autoregulation by effective recanalization, and thus minimize ischemic core and salvage peri-infarct region where autoregulation was proven to be disturbed (2). Data from animal models demonstrated that wild-type tissue plasminogen activator, even when administered a clinically relevant time frame after stroke, disrupted CBF autoregulation by impairing vasodilation (22). Further research on the underlying mechanism implied that tissue plasminogen activator could have detrimental effects on autoregulation by aggravating overactivity of N-methyl-d-aspartate receptors, which was the key element in coupling local metabolism to CBF (23). To our limited knowledge, only one clinical study reported dCA of AIS after rt-PA thrombolysis. The results illustrated that in middle cerebral artery occlusion patients with successful thrombolysis and small infarction, dCA was not altered as compared to that in minor stroke patients who did not receive thrombolysis and healthy controls; this finding proved that at least there was no additional adverse effects of the administration of rt-PA (20). In our study cohort, dCA of patients who received intravenous rt-PA thrombolysis was relatively preserved on the ipsilateral side than in those who did not receive, and it was still a significant factor in the multivariate regression model. The relatively preserved dCA on the affected hemisphere was probably because of possible recanalization of occlusive artery or even arteriole after thrombolysis. But there was still a confounding factor involved that patients who receiving rt-PA thrombolysis usually had a shorter onset-to-therapy time, and therefore may receive timely medical care and medication from the first hours of stroke. Thus, further well-designed clinical trials are required to better understand the effect of rt-PA thrombolysis on dCA from clinical aspects.

Serum uric acid level was the only positive variable among the various laboratory results that presented an interesting relationship between uric acid and dCA. In our cohort of AIS patients with a relatively higher average uric acid level on admission, we found that higher uric acid level was correlated with disrupted autoregulation bilaterally both on acute and subacute phase and even had the prognostic value for impaired dCA independent of other clinical factors. Although it hardly demonstrated a causal relationship between uric acid and dCA, it was still an interesting finding that uric acid may potentially become a therapeutic target for future investigations. Previous studies assessing the association between uric acid levels and clinical outcome reported conflicting results (24–26). Although it was demonstrated that uric acid was a possible neuroprotective factor owing to its antioxidant effect in the face of acute ischemia injury (27), the effect on cerebral hemodynamic has not been investigated previously. Therefore, further research is urgently required from both the clinical and its underlying mechanism aspects to verify our findings before an appropriate serum uric acid level can be recommended with adequate evidence as guidelines.

It was supported by previous studies that an impaired dCA played an important role in leading to adverse clinical events including larger stroke volume, cerebral edema, hemorrhagic transformation, and also poor clinical outcomes (3–5). Yet, whether dCA has the potential prognostic value for clinical outcomes has not been frequently put forward. A cohort of 45 AIS patients was collected and dCA was analyzed to investigate the relationship between autoregulation and clinical outcomes. The correlation was detected between dCA parameters and mRS, but in following multivariate analyses, dCA did not remain significant predictor for clinical outcomes (3). Recent study from Castro et al. demonstrated a comparable conclusion that early effective autoregulation was associated with better neurological outcome. They not only proved dCA parameter as an independent predictor but also found out the cut-off point, PD >37° for predicting neurological independence at 3 months (5). In line with their results, the cut-off value in our study was 35.37° and the difference between Castro's and our study was that they chose the PD value during the first 6 h after symptoms onset while in our study significant PD value for predicting outcomes was in subacute phase. Those observation indicated autoregulation parameter PD may be a stable prognostic value for clinical outcomes.

The main limitation of the present study is its methodological accuracy with lower spatial resolution of TCD. In view of its non-invasive bedside assessment with high temporal resolution, it presently remains the most prevalent approach in clinical dCA studies. Moreover, although providing the most elaborate clinical information possible, the relatively small sample size might not be enough to detect some potential differences within groups or variables. In addition, the strict inclusion criteria led to lower NIHSS score and disproportional subtypes of our subjects. Therefore, whether our preliminary results could apply to general stroke population is required to be confirmed with a larger study cohort.

## Conclusion

DCA was sustained to be bilaterally impaired in the acute and even subacute phase after AIS. Our study may advance knowledge of the relationship between dCA and clinical factors. Patients who receiving rt-PA thrombolysis tended to have a better dCA in acute phase. Increasing age, subtype of LAA, and higher uric acid level had prognostic value for disturbed autoregulation. A relatively preserved dCA may predict good clinical outcomes.

## Author contributions

HM, ZG, XS and YY designed the study. HJ and XY performed data collection, and HM, ZG, JL and SL performed the dCA analyses. PZ performed the statistical analysis. All authors interpreted the data. HM wrote the manuscript. All authors had full access to the data and helped in critically revising the manuscript before reviewing and approving the final version.

### Conflict of interest statement

The authors declare that the research was conducted in the absence of any commercial or financial relationships that could be construed as a potential conflict of interest.
